# Sexual transmission of *Anopheles gambiae* densovirus (AgDNV) leads to disseminated infection in mated females

**DOI:** 10.1186/s13071-022-05341-4

**Published:** 2022-06-20

**Authors:** Kristine L. Werling, Rebecca M. Johnson, Hillery C. Metz, Jason L. Rasgon

**Affiliations:** 1 Department of Entomology, Pennsylvania State University, University Park, PA USA; 2 Center for Infectious Disease Dynamics, Pennsylvania State University, University Park, PA USA; 3 The Huck Institutes of the Life Sciences, Pennsylvania State University, University Park, PA USA; 4 Present Address: Department of Environmental Sciences, The Connecticut Agricultural Experiment Station, New Haven, CT 06504 USA

**Keywords:** Insect specific virus, Densovirus, *Anopheles gambiae*, Paratransgenesis, Male releases

## Abstract

**Background:**

*Anopheles gambiae* densovirus (AgDNV) is an insect-specific, single-stranded DNA virus that infects *An. gambiae *sensu stricto (s.s.), the major mosquito species responsible for transmitting malaria parasites throughout sub-Saharan Africa. AgDNV is a benign virus that is very specific to its mosquito host and therefore has the potential to serve as a vector control tool via paratransgenesis (genetic modification of mosquito symbionts) to limit transmission of human pathogens. Prior to being engineered into a control tool, the natural transmission dynamics of AgDNV between *An. gambiae* mosquitoes needs to be fully understood. Additionally, improved knowledge of AgDNV infection in male mosquitoes is needed. In the study presented here, we examined the tissue tropism of AgDNV in the male reproductive tract and investigated both venereal and vertical transmission dynamics of the virus.

**Methods:**

*Anopheles gambiae* s.s. adult males were infected with AgDNV via microinjection, and reproductive tissues were collected and assayed for AgDNV using qPCR. Next, uninfected females were introduced to AgDNV-infected or control males and, after several nights of mating, both the spermatheca and female carcass were assessed for venereally transmitted AgDNV. Finally, F1 offspring of this cross were collected and assayed to quantify vertical transmission of the virus.

**Results:**

AgDNV infected the reproductive tract of male mosquitoes, including the testes and male accessory glands, without affecting mating rates. AgDNV-infected males venereally transmitted the virus to females, and these venereally infected females developed disseminated infection throughout the body. However, AgDNV was not vertically transmitted to the F1 offspring of this cross.

**Conclusions:**

Infected male releases could be an effective strategy to introduce AgDNV-based paratransgenic tools into naïve populations of* An. gambiae* s.s. females.

**Graphical Abstract:**

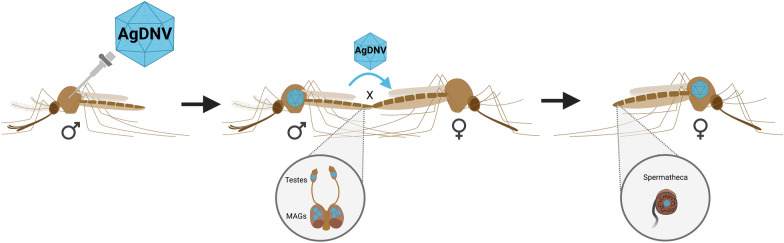

## Background

*Anopheles gambiae* densovirus (AgDNV) is an insect-specific virus (ISV) that efficiently infects *An. gambiae *sensu stricto (s.s.) mosquitoes [1, 2], one of the major vectors of malaria-causing parasites. This non-enveloped, single-stranded DNA virus belongs to the *Parvoviridae* family and *Brevidensovirus* genus and has a compact genome of approximately 4.1 kb [1]. First identified in an *An. gambiae* cell line (Sua5B) [1], AgDNV is specific to* An. gambiae* s.s. mosquitoes [2] and can also likely infect the sibling species *Anopheles coluzzii* (due to the mixed origin of Sua5B cells). It replicates only minimally in closely related *Anopheles arabiensis* and cannot replicate in more distantly related *Anopheles* or other mosquito genera [2]. Importantly, AgDNV is also unable to infect vertebrates [2]. Due to this host specificity, ISVs like AgDNV offer a promising new avenue for targeted biological control of individual mosquito species and the pathogens they transmit [3–5]. In its natural* An. gambiae* s.s. host (hereinafter referred to as *An. gambiae*)*,* AgDNV can infect both sexes and all developmental stages, replicating particularly well in adults post-emergence [6, 7]. Notably, AgDNV is a benign virus that does not cause mortality or even a marked transcriptional response in its mosquito host [8]. This is in contrast to several related mosquito-specific densoviruses that infect *Aedes* mosquitoes with high lethality [9–11]. While cytotoxic densoviruses can be harnessed to make novel biopesticides [5], as was done with the *Aedes aegypti* densovirus (AaeDNV) [12], the benign nature of AgDNV is highly advantageous for another vector control approach known as paratransgenesis, or the genetic modification of symbionts with the goal of decreasing pathogen transmission. Specifically, AgDNV could be genetically modified to encode a specific effector that, upon delivery and expression in its *An. gambiae* host, targets either the mosquito or the pathogens they transmit to ultimately decrease the incidence of mosquito-borne diseases like malaria. The potential use of AgDNV for paratransgenesis has previously been demonstrated [1, 7, 13], but for an AgDNV-based tool to be effective in field settings, we first need a complete understanding of AgDNV transmission dynamics between *An. gambiae* hosts. This information is critical for determining the best application and deployment strategy for an AgDNV-based vector control tool.

A previous study found that AgDNV can be venereally transmitted from infected males to the spermatheca of females post-mating [6], yet it is not known if venereally transmitted AgDNV remains contained to the spermatheca or if it can also lead to disseminated infection in females. This information is important for determining whether infected male releases could be a viable method for introducing AgDNV-based tools into field populations of female *An. gambiae*. As only female mosquitoes blood feed and transmit disease-causing pathogens, they are the main target of vector control programs, and as such, male mosquitoes can be safely released into a population without directly affecting pathogen transmission. Modified male releases is an approach that has been successful for certain *Aedes aegypti* control programs using either the endosymbiont bacterium *Wolbachia* [14] or transgenic male mosquitoes [15, 16]. In addition to venereal transmission, AgDNV can also be vertically transmitted from mother to offspring when the mother is infected during larval stages [1], but it is not yet known if mothers infected venereally can subsequently transmit AgDNV to the next generation. This knowledge would reveal if AgDNV-based tools could disseminate trans-generationally into a population following infected male releases.

We also do not know the tissue tropism of AgDNV in male mosquitoes, particularly in their reproductive tissues where infection could impact mating success. Notably, *plugin* is one of the few genes that is differentially expressed in AgDNV-infected mosquitoes [8]. It encodes a fundamental component of the male mating plug, a coagulated mass of seminal secretions transferred to females during copulation that is essential for proper sperm storage in females [17]. Given that AgDNV influences the expression of this key mating factor, assessing whether AgDNV infection influences male mating rates is also an urgent question, as this could impinge on the success of AgDNV-based tools.

In the study reported here, we addressed these questions by examining AgDNV tropism and transmission dynamics in mosquitoes infected in the laboratory. We found that AgDNV spreads venereally from infected males to naïve females, such that females develop disseminated infection; however, AgDNV is not subsequently transmitted vertically to F1 offspring. Our findings demonstrate that AgDNV holds promise as a tool for paratransgenesis in *An. gambiae*, whereby infected male releases could be utilized for dissemination into field populations.

## Methods

### Production of AgDNV (GenBank: EU233812.1)

Moss55 cells, which naturally lack wild-type AgDNV [1], were transfected with a plasmid containing the complete wild-type genome of AgDNV, as previously described [1, 6, 13]. Briefly, 6-well plates were seeded with approximately 3 × 10^6^ cells per well. The following day, transfections were performed using Lipofectamine LTX with Plus Reagent (Thermo Fisher Scientific, Waltham, MA, USA) and 2.5 µg of AgDNV-containing plasmid (pAgDNV) per well. Three days after transfection, AgDNV virus was harvested as follows: cells were washed and re-suspended in phosphate-buffered saline (PBS), lysed via 3 freeze/thaw cycles (alternating − 80 °C and room temperature) and then spun down to remove cell debris. Supernatant was collected, aliquoted and stored at − 80 °C. This supernatant served as the AgDNV viral inoculum for all subsequent experiments.

### Mosquito rearing

*Anopheles gambiae* s.s. (Keele strain) were reared at 27 °C and approximately 80% humidity under a 12/12-h light/dark cycle. Larvae were fed ground Tetramin flakes (Tetra Werke, Melle, Germany) daily. At the pupal stage, males and females were separated to maintain virgin adults. Adults were provided 10% sucrose solution throughout.

### Microinjections

At 2–3 days post-emergence, virgin males were inter-thoracically injected with approximately 200 nl of either AgDNV viral stock or PBS (negative control) using a Nanoject II Auto-Nanoliter Injector (Drummond Scientific Co., Broomall, PA, USA) and glass capillary needles back-filled with mineral oil. Mosquitoes injected with AgDNV each received an estimated 10^6^ viral genome equivalents (vge). Mosquitoes were cold-anesthetized using ice and a cold-block during injections, and immediately placed to recover at room temperature after being injected.

### Mating

When AgDNV- and PBS-injected male mosquitoes reached 14 days post-injection (dpi), virgin females were introduced into their cages using a mouth aspirator (John W Hock Co., Gainesville, FL, USA). Females were added in a 1:2 ratio to males, and these mixed cages were left for 3 nights to allow for mating, following which the mixed sex cage was briefly chilled at 4 °C to anesthetize the mosquitoes. Males and females were then separated over ice using a paintbrush and forceps and transferred to new cages.

### Blood-feeding and oviposition for F1 offspring

Female mosquitoes were provided with a blood meal at 1 and 7 days post-mating. Blood-feeding was achieved using a 37 °C water bath, glass membrane feeder, and anonymous human blood sourced from BioIVT (Westbury, NY, USA). Two days after each blood-feeding, females were provided with an oviposition site (petri dish with wet cotton and filter paper). Eggs were collected 3 days later and immediately hatched and reared as described above.

### Tissue collections

Dissected tissues were either stored dry (carcasses) or collected in 50–100 µl of PBS (spermathecas, testes, male accessory glands [MAGs]) and stored at − 80 °C until DNA extraction.

#### Males

Male reproductive tissues (testes and MAGs) were collected in pools of 12, while male carcasses (body without reproductive tract) were analyzed individually. Males were analyzed at 11, 13, 15 and 18 dpi.

#### Females

Female mating status was determined by visual examination of the spermatheca for the presence or absence of sperm. Only mated females were further processed for AgDNV quantification. Spermathecas were pooled into groups of 5–13 tissues, while female carcasses (body without spermatheca) were analyzed individually. Females were analyzed at 8–14 days post-mating (estimated, because exact time of mating was not known).

#### F1 offspring

F1 offspring were collected as whole mosquitoes, and both males and females were analyzed in groups of four to five individuals. F1 adults were maintained in a mixed sex cage until collection.

### DNA extraction from mosquito tissues

Tissues were first homogenized using either sterile pellet pestles (Fisherbrand™, Thermo Fisher Scientific) and an electric pestle grinder (Kimble®; Dwk Life Sciences LLC, Millville, NJ, USA) (spermathecas, testes, MAGs), or stainless steel beads (OPS Diagnostics, Lebanon, NJ, USA) and the TissueLyser II small bead mill (Qiagen, Hilden, Germany) (carcasses). After homogenization, TL buffer (Omega Bio-Tek Inc., Norcross, GA, USA) was added to reach a final volume of 200 ul. DNA was then extracted from the samples using the E.Z.N.A Tissue DNA kit (Omega Bio-Tek) according to the manufacturer’s instructions. Proteinase K incubation was carried out for 60 min at 55 °C in a water bath with vortexing every 15–20 min, and final samples were eluted twice with 50 ul elution buffer warmed to 70 °C. Sample quantity and quality were checked using a Nanodrop spectrophotometer (Thermo Fisher Scientific) before proceeding to quantitative PCR (qPCR).

### Quantification of AgDNV

#### Quantification of AgDNV viral stock produced from transfected cells

One aliquot of virus stock was removed from storage at − 80 °C and thawed on ice. Using Turbo™ DNase (Invitrogen™, Thermo Fisher Scientific) the sample was treated to remove any remaining plasmid DNA that may have been left over from the transfection and would interfere with viral quantification. Next, viral DNA was extracted from the stock using the E.Z.N.A Tissue DNA kit (Omega Bio-Tek) according to the manufacturer’s instructions for cultured cells, starting with Proteinase K treatment. qPCR was then run on a Roto-Gene Q real-time cycler (Qiagen) using the PerfeCTa SYBR Green Master Mix (Quantabio, Beverly, MA, USA) and primers targeting the viral protein (VP) gene on the AgDNV genome. Samples were run alongside a standard curve, consisting of a pAgDNV dilution series, to allow for quantification of vge per milliliter. VP primer sequences were the same as those used in a previous study [22]: 5′-GGC ATC AAT GTG GGA CCA AG-3′ (forward) and 5′-CCG TTA GCA AGC GTT GTC TG-3′ (reverse). qPCR cycling conditions were an initial cycle of 95 °C for 5 min, followed by 40 cycles of 95 °C for 10 s, then by a data acquisition step of 60 °C for 30 s; a melt curve analysis was run at the end of cycling by going from 72 °C to 95 °C with 5 s per step.

#### Quantifying AgDNV from mosquito tissues

Following DNA extraction, samples were analyzed by qPCR, as described above, except that a pAgDNV standard curve was not run. Instead, primers targeting the host ribosomal protein gene S7 were run alongside primers targeting the viral VP gene. Relative AgDNV titers were then determined by normalizing VP quantification (viral genomes) to* S7* quantification (host genomes). This step normalizes for differences in the amount of total DNA obtained from different tissue types (e.g. testes vs. carcass). S7 primer sequences were: 5’-AAG GGT TGC GTG CTA GTG AA-3’ (forward) and 5’-TAA CGG CTT TTC TGC GTC CA-3’ (reverse). AgDNV dissemination rates in mated female carcasses (see text associated with Fig. 2b) were estimated by using the highest detectable VP/S7 value found in control female carcasses (0.0000845 VP/S7) as the cut-off for determining AgDNV-positive (*n* = 45, 90%) or AgDNV-negative samples (*n* = 5, 10%).

### Statistical analyses

Data for relative AgDNV titers were first tested for normality (Kolmogorov–Smirnov, D’Agostino & Pearson) and then, where appropriate, log [Ln(y)] transformed to achieve normality. When analyzing two groups, unpaired Student t-tests were used for data with equal variances, and Welch t-tests were used for data with unequal variances. If analyzing more than two groups, one-way analysis of variance (ANOVA) (equal variances) or Brown-Forsythe/Welch ANOVA (unequal variances) was used with either Sidak’s or Dunnett’s multiple testing correction, respectively. Mating and survival rates were analyzed using Fisher’s exact tests. Statistical tests are also indicated in the figure and table legends. A *P*-value cut-off of < 0.05 was used to determine significance. Analyses were performed with either GraphPad Prism (GraphPad Software Inc., San Diego, CA, USA) or JMP software (SAS Institute, Cary, NC, USA).

## Results

### AgDNV infects male reproductive tissues without affecting mating rates

**Fig. 1:**
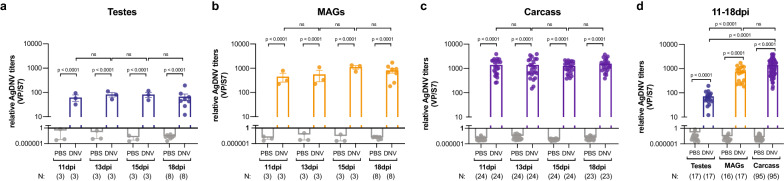
AgDNV infects the reproductive tract of *Anopheles gambiae* males. **a**–**c** Males were injected with either AgDNV (DNV on figure) or PBS (control).** a**–**c** At 11, 13, 15 and 18 dpi, AgDNV-treated males have infected: **a** testes [Ln(y) transformed, one-way ANOVA, Sidak’s correction], **b** MAGs [Ln(y) transformed, one-way ANOVA, Sidak’s correction], **c** carcass [Ln(y) transformed, Brown-Forsythe/Welch ANOVA, Dunnett’s correction]. However, viral titers do not change over time in any tissue. **d** Independent of time (including 11-18 dpi), AgDNV titers in the MAGs and carcass are significantly higher than those in the testes [Ln(y) transformed, Brown-Forsythe/Welch ANOVA, Dunnett’s correction]. NS indicates *P*-value > 0.05. Throughout, for testes and MAGs, each point represents a pool of 12 tissues; for carcasses, each point represents an individual carcass with the reproductive tract removed. Viral genomes are normalized to host genomes (S7). Abbreviations: AgDNV, *Anopheles gambiae* densovirus; ANOVA, analysis of variance; dpi, days post-injection; Ln(y), log transformed; MAGs, male accessory glands; PBS, phosphate-buffered saline; S7, host ribosomal protein gene; VP, viral protein

To determine the tissue tropism and infection dynamics of AgDNV in the reproductive tract of *An. gambiae* males, we infected young adult males with AgDNV via inter-thoracic microinjections and then assessed viral titers at four time points, relative to PBS-injected controls. We found that AgDNV infects both major male reproductive tissues, namely the testes (Fig. 1a) and the male accessory glands (MAGs) (Fig. 1b), as well as the remainder of the body (hereafter referred to as the carcass) (Fig. 1c). AgDNV was detectable in all tissues at 11, 13, 15, and 18 dpi, yet within each tissue type, relative viral titers (i.e. normalized to host genome) did not differ significantly between time points (Fig. 1a–c). Independent of time post-injection, we found significantly higher relative AgDNV titers in the MAGs compared to the testes (Fig. 1d), with MAG titers comparable to those of the carcass. We summarize these results as showing that both components of the male reproductive tract are susceptible to infection, but that AgDNV likely replicates more efficiently in the MAGs than in the testes.

To assess whether AgDNV infection in male mosquitoes affects their ability to mate, we introduced uninfected, virgin females into cages with either AgDNV- or PBS-injected males. After 3 nights of mating, females were dissected and mating status was determined by the presence or absence of sperm in the spermatheca. We found no difference in the proportion of mated females between those exposed to AgDNV-injected males and those exposed to PBS-injected males (Table 1), indicating that AgDNV infection does not significantly hinder male mating ability. Additionally, we found no difference in the survival of AgDNV-injected males compared to controls when assessed at 18 dpi (Table 2), supporting previous findings that AgDNV infection does not affect longevity of its *An. gambiae* host [8].

**Table 1:** Mating rates between virgin females with either AgDNV- or PBS-injected male *Anopheles gambiae*

Mating rates^a^	PBS-injected males		AgDNV-injected male	
	Mated	Unmated	Mated	Unmated
P	29%	71%	36%	64%
N	46	111	63	114
Total number	157		177	

The mating rate between PBS-injected males and AgDNV-injected males did not differ (Fisher’s exact test, *P* = 0.2432), as determined by the number of females that were inseminated after being left with males for 3 nights

*AgDNV Anopheles gambiae* densovirus,* PBS* phosphate-buffered saline

^a^N = number of female mosquitoes, either with or without sperm present in the spermatheca (classified as mated or unmated, respectively). P = percentage of female mosquitoes that are mated or unmated

**Table 2:** Male *An. gambiae* survival rates

Survival rates^a^	PBS-injected males		AgDNV-injected males	
	Alive	Dead	Alive	Dead
P	71%	29%	75%	25%
N	371	151	428	141
Total number	522		569	

The survival of PBS-injected males and AgDNV-injected males (Fisher’s exact test, *P* = 0.1322) did not differ when assessed at 18 dpays post-injection

^a^N = number of male mosquitoes that are either alive or dead. P = percentage of male mosquitoes that are either alive or dead

### *An. gambiae* females develop disseminated infection after mating with AgDNV-infected males

**Fig. 2:**
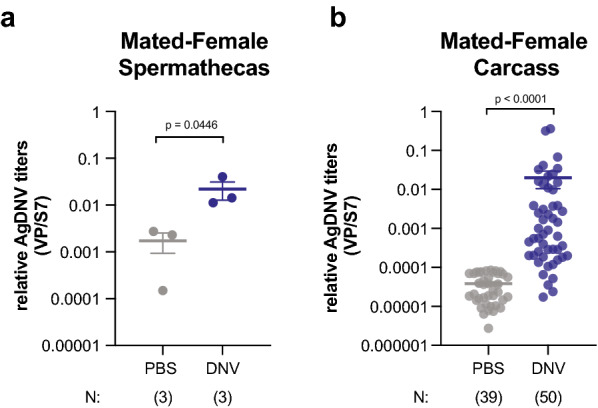
Venereally transmitted AgDNV causes disseminated infection in mated females. **a**, **b** Females mated to AgDNV-injected males have** a** Detectable AgDNV in the spermatheca [Ln(y) transformed, unpaired Student’s t-test; each point = pool of 5–13 tissues], **b** disseminated AgDNV infection in the carcass [Ln(y) transformed, Welch’s t-test, each point = single carcass with spermatheca removed], when analyzed 8–14 days post-mating and relative to females mated to PBS-treated mates. Viral genomes are normalized to host genomes (S7)

Authors of previous studies reported that AgDNV is venereally transmitted from infected males to the spermatheca of mated females [6]. To test whether sexually-transmitted AgDNV can also lead to disseminated infection in mated females, we again allowed uninfected, virgin females to mate with either AgDNV- or PBS-injected males. After mating, the females were separated and maintained for 8–14 days before being analyzed. As expected, we detected AgDNV in the spermatheca of females mated to infected males (Fig. 2a). We also found significant levels of AgDNV in the female carcass (Fig. 2b). Although AgDNV titers were variable, an estimated 90% of mated females (*n* = 45) developed disseminated infection after exposure to AgDNV-infected males (see Methods).

### Females venereally infected with AgDNV fail to vertically transmit to F1 progeny

**Fig. 3:**
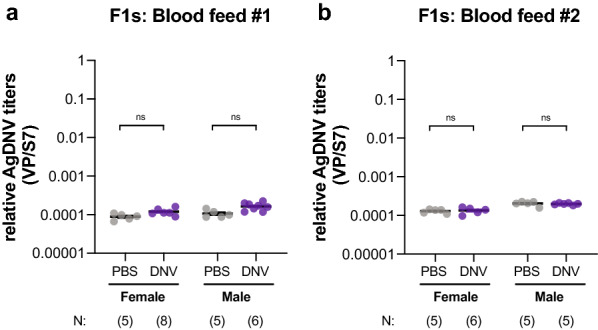
Sexually transmitted AgDNV is not vertically transmitted from mother to offspring. **a**, **b** Females mated to AgDNV-infected males were provided a blood meal and the resulting F1 offspring were reared to adults. AgDNV was not detectable in male or female F1 progeny (whole bodies) from either a first blood-feeding (**a**; one-way ANOVA, Sidak’s correction, each point = pool of 4–5 whole mosquitoes), or a second blood-feeding (**b**; one-way ANOVA, Sidak’s correction, each point = pool of 4–5 whole mosquitoes), as compared to F1 offspring from PBS-injected fathers. NS indicates *P*-value > 0.05. Viral genomes are normalized to host genomes (S7)

We next assessed whether females sexually infected with AgDNV can vertically transmit the virus to their offspring. Specifically, after females were mated to AgDNV- or PBS-injected males, they were provided a blood meal and an oviposition site. Oviposited eggs were reared to adulthood, and F1 male and female offspring were pooled and assessed for virus. Across all samples tested, AgDNV was not detectable in F1 progeny (whole bodies) from either a first or second blood-feeding (Fig. 3a, b). These findings reveal that even though venereally transmitted AgDNV leads to disseminated infection in mated females, they do not vertically transmit the infection to their offspring.

## Discussion

Here we report that venereal transmission of AgDNV leads to disseminated infection in *An. gambiae* females. This finding reveals that infected male releases could be an effective way to introduce AgDNV-based paratransgenic tools into field populations of *An. gambiae* females. More specifically, released males harboring a modified AgDNV would mate with wild females and venereally transmit the virus to them. Then, as AgDNV titers build up in the female post-mating, a viral-encoded effector could interfere with the ability of the mosquito to transmit pathogens like malaria parasites. Male releases have already been shown to be a safe and effective way to deploy biocontrol agents into mosquito populations [14–16], largely because male mosquitoes do not blood feed and thus are not responsible for pathogen transmission. Treatment of larval breeding sites is another means by which ISV-based control tools could be deployed in the field, as was done for the AaeDNV-based larvicide [5, 12], but this approach poses significant challenges for *An. gambiae*. *Anopheles gambiae* females often lay their eggs in small temporary breeding sites produced by rain fall, making large-scale application of larvae-targeting tools generally impractical for this mosquito species in the field setting [18]. Therefore, infected male releases offer a more logistically feasible method to introduce AgDNV-based control tools into wild *An. gambiae* populations.

For infected male releases to be successful, AgDNV must not only be venereally transmitted, but AgDNV-infected males must also be fit and able to mate at rates competitive with wild males. In the present study, we found that AgDNV infection in males does not affect mating rates, a good first indication of reproductive fitness. To fully assess mating competitiveness for field releases, mate-choice experiments with wild-type mosquitoes in a semi-field environment would still be needed. We also assessed the tissue tropism of AgDNV in male mosquitoes and found that both the testes and the MAGs become infected, indicating that venereally transmitted virus could be transferred via sperm (produced in the testes), the mating-plug (produced from the MAGs) or both. After mating, sperm is stored in the female spermatheca and the mating-plug is digested in the female atrium (uterus) [19]; since we observed AgDNV in both the spermatheca and the carcass of mated females, it is likely that both components contribute to sexual transmission of AgDNV. While we observed AgDNV in all spermatheca pools tested (Fig. 2a), due to the pooling of tissues, we are unable to determine the exact rate of venereal transmission. In the future, paired mating assays, such as mating captures, coupled with individual tissue analyses could provide this information as well as enable determination of how variation in male viral titers influences transmission and dissemination in mated females.

We also found no difference in AgDNV titers across time in any of the male tissues assessed, demonstrating that peak viral titers are achieved by 11 dpi, in agreement with the results of a previous study [6], which found that adult AgDNV titers increase up to ~12 dpi.

In this study, we used the microinjection method to infect male mosquitoes with AgDNV. This approach allowed for controlled and reproducible delivery of inoculating AgDNV doses for the characterization of infection and transmission dynamics. However, microinjections are laborious and would be an impractical strategy to infect male mosquitoes on a large scale, as would be needed for an AgDNV-based male release program. Therefore, although logistically challenging for field applications, treatment of larval breeding water with AgDNV could be an effective way of mass-producing infected *An. gambiae* in the laboratory. In fact, it was previously shown that adult mosquitoes can become infected with AgDNV following the exposure of larvae to AgDNV-treated water [1, 7]. This infection method would need to be further developed for operational purposes, but previous data suggest that when mosquitoes are infected via larval exposure, adult AgDNV titers are similar to those achieved by injection [6, 7] (Fig. 1c). Notably, viral titers may even reach their peak more quickly in adults that were infected as larvae [7] as compared to those infected by injection [6] (7 vs. 12 days post-emergence, respectively). This means that larval exposure to AgDNV could allow for the release of younger infected males, furthering the feasibility of this approach over microinjections for operational purposes. Data from females suggest that AgDNV tissue tropism is consistent between infection routes [1, 13], yet male tissue tropism and venereal transmission dynamics following larval exposure still need to be tested. This information would help optimize the larval exposure method in the laboratory to maximize male infection and venereal transmission rates for any future AgDNV-based male release programs.

A previous study showed that AgDNV is transferred to the female spermatheca immediately post-mating [6], but the authors of that study did not detect significant dissemination in the female carcass. This discrepancy likely arises due to low statistical power in the previous study [6]: elevated DNV was detected in the carcass but it was not statistically significant. In addition, females in this study [6] were assessed immediately after mating, whereas in the present study we assayed for AgDNV in females 8–14 days post-mating, allowing time for the virus to replicate. Given that AgDNV titers are known to rise gradually in adult females [6, 7], it is not surprising that it took a few days for AgDNV titers to increase to significant levels in mated females. In fact, the gradual increase in AgDNV titers over time is one reason this ISV has been proposed as a candidate for a “late-life-acting” insecticide [7, 20]. In the present study, we did not assess any female fitness parameters following venereally acquired AgDNV infection because previous work [8] found that mosquitoes directly infected with high doses of AgDNV do not exhibit reduced fitness or even a marked transcriptional response. We do not expect venereally transmitted AgDNV to affect female fitness, but prior to implementation of AgDNV-based tools, this possibility would need to be tested.

Although venereally transmitted AgDNV produces disseminated infection in females, we found no evidence of vertical transmission from these females. Interestingly, previous studies demonstrated vertical transmission of AgDNV when both male and female mosquitoes were directly infected by exposure to virus during larval development [1]. There are several possible reasons why we did not observe vertical transmission in our experiments. First, although AgDNV was present in the female body, it may not have infected the ovaries/oocytes. Secondly, AgDNV present in the spermatheca may not be directly associated with sperm cells and therefore not likely to enter the egg during fertilization. Finally, it is possible that AgDNV titers simply were not high enough for vertical transmission to occur in these mosquitoes. Without vertical transmission, an AgDNV-based vector control tool would not spread trans-generationally following infected male releases. This means that a greater frequency of male releases would be required to have an impact on wild *An. gambiae* populations; on the other hand, this feature may be advantageous from a public acceptance stand-point, because the virus and its encoded effectors would not be heritable across multiple generations. This feature would give more user control over the application of these paratransgenic tools.

Our studies here focused exclusively on the infection and transmission dynamics of wild-type AgDNV. This contrasts with several previous studies of AgDNV-based paratransgenesis that have largely focused on a two-virus helper/transducer system (consisting of one wild-type AgDNV and one defective, GFP-expressing AgDNV) [1, 6, 13]. While this helper/transducer approach offers a larger capacity for transgenic cargo, it is far more practical to utilize a single, self-replicating transgenic virus for application in field settings. Viral packaging efficiency limits the size of transgenes that can be encoded in a single AgDNV vector, but small anti-pathogenic effectors or short-hairpin RNAs targeting host processes could be expressed under an endogenous promoter to limit the mosquito’s ability to transmit pathogens. Further engineering and testing are still needed to identify and validate the best effectors for this system.

Finally, in addition to offering paratransgenic tools, ISVs can also have natural pathogen-blocking effects [21]. For example, AgDNV has an inhibitory effect on the emerging arbovirus Mayaro virus [22], which can be transmitted by *Anopheles* [23]. Wild-type AgDNV may also inhibit additional pathogens, such as the malaria parasite, although this remains to be explored. Together, our findings underscore the importance of basic research into the dynamics of wild-type ISVs such as AgDNV, which hold promise for both basic and applied purposes.

## Conclusions

These findings reveal that if AgDNV is developed into a paratransgenic tool for *Anopheles gambiae* mosquitoes, it could be potentially deployed in the field via infected male releases. *Anopheles gambiae* spreads more fatal infections to humans than any other vector, yet to date we still lack genetic tools for targeting this species and AgDNV may fill this gap.

## Data Availability

All data generated or analyzed during this study are included in this published article.

## Abbreviations

AgDNV: *Anopheles gambiae* densovirus
dpi: Days post-injection
ISV: Insect-specific virus
MAGs: Male accessory glands
pAgDNV: Plasmid containing complete AgDNV genome
S7: Host ribosomal protein gene
vge: Viral genome equivalents
VP: Viral protein (gene)
